# Enhancement of silicon sub-bandgap photodetection by helium-ion implantation

**DOI:** 10.1007/s12200-023-00096-x

**Published:** 2023-12-06

**Authors:** Zhao Wang, Xiaolei Wen, Kai Zou, Yun Meng, Jinwei Zeng, Jian Wang, Huan Hu, Xiaolong Hu

**Affiliations:** 1 School of Precision Instrument and Optoelectronic Engineering, Tianjin University, Tianjin, 300072 China; 2 Key Laboratory of Optoelectronic Information Science and Technology, Ministry of Education, Tianjin, 300072 China; 3 Center for Micro and Nanoscale Research and Fabrication, University of Science and Technology of China, Hefei, 230026 China; 4 Wuhan National Laboratory for Optoelectronics, Huazhong University of Science and Technology, Wuhan, 430074 China; 5 Optics Valley Laboratory, Wuhan, 430074 China; 6 ZJUI Institute, International Campus, Zhejiang University, Haining, 311400 China; 7 State Key Laboratory of Fluidic Power & Mechanical Systems, Zhejiang University, Hangzhou, 310027 China

**Keywords:** Sub-bandgap optical absorption, Helium-ion implantation, Silicon photodetector, Non-invasive photodetection

## Abstract

Silicon sub-bandgap photodetectors can detect light at the infrared telecommunication wavelengths but with relatively weak photo-response. In this work, we demonstrate the enhancement of sub-bandgap photodetection in silicon by helium-ion implantation, without affecting the transparency that is an important beneficial feature of this type of photodetectors. With an implantation dose of 1 × 10^13^ ions/cm^2^, the minimal detectable optical power can be improved from − 33.2 to − 63.1 dBm, or, by 29.9 dB, at the wavelength of 1550 nm, and the photo-response at the same optical power (− 10 dBm) can be enhanced by approximately 18.8 dB. Our work provides a method for strategically modifying the intrinsic trade-off between transparency and strong photo-responses of this type of photodetectors.

**Graphical Abstract:**

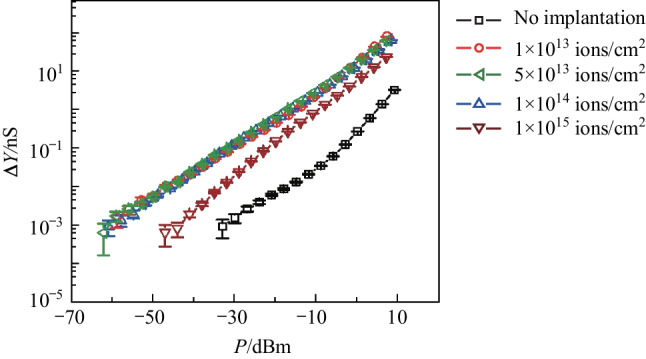

## Introduction

Harnessing sub-bandgap optical absorption in semiconductors can extend the photo-response spectra of photodetectors beyond the long-wavelength limit [1–7]. One of the specific applications of sub-bandgap optical absorption is the contactless integrated photonic probe (CLIPPs). These devices were made to non-invasively monitor the changes and fluctuations of optical power in silicon waveguides and on more sophisticated photonic circuits [1], and they were combined with feedback systems for feedback control of photonic integrated circuits [2]. By optimizing the device structure [3] or reducing noise [4], the sensitivity of CLIPPs has been further improved. Additionally, normal-incidence silicon photodetectors that are analogous to CLIPPs and that can detect free-space light at the infrared telecommunication wavelengths have been demonstrated [5, 6]; four-quadrant photodetectors have been designed and made to track the positions and deflections of light beams in free space [7].

One important feature of these photodetectors is their non-invasiveness, or, transparency, and in this respect, they are quite different from commonly used photodetectors. The fundamental reason for this feature is that sub-bandgap optical absorption is weak, compared with inter-band optical absorption. This benefit leads to many applications; however, it also means that these photodetectors show low sensitivity and limited photo-responsivity, which may not work well, or may fail, when the to-be-detected light is faint. Indeed, non-invasiveness, or transparency, and strong photo-response are trade-off behaviors.

This trade-off can be mitigated by defect-state engineering [8, 9]. Strategically increasing the density of defect states within the bandgap by ion implantation can enhance the photo-response, without noticeably sacrificing the non-invasiveness or transparency. In Ref. [10], researchers implanted helium ions by ion implanter to silicon waveguides to fabricate in-line photodetectors that can work in the wavelength range between 1400 and 1590 nm.

In a broader context, ion implantation has important impact on the performance of photodetectors in general. Researchers have implanted B ions into silicon-based mesa heterojunction photodetectors and found that ion implantation significantly improved the photoelectric properties of devices [11]; other researchers have implanted Zr ions into CsPbBr_3_ perovskite single-crystal photodetectors to enhance the performances of the devices [12]; others have studied the photoelectrical performance of Schottky photodetectors made of β-Ga_2_O_3_ implanted with Mg ions [13]; some have implanted Argon ions to silicon Schottky-type photodetectors to enhance the responsivity at infrared 1.31 and 1.55 μm [14]. Additionally, ion implantation has been found to have impact on the nonlinear properties of silicon [15].

In this letter, we report use of a helium-ion microscope to implant helium ions into normal-incidence silicon sub-bandgap photodetectors [5]. The helium-ion microscope offers the capability of implanting with a super small beam spot of ~ 0.5 nm and has demonstrated promising applications in modifying material properties for superconducting junctions [16] and memristors [17]. Here we report the experiment on applying helium-ion beam implantation to enhance the photo-response and sensitivity beyond the long-wavelength limit without affecting the transparency of the sub-bandgap photodetector. Our experimental results show that by implanting helium ions with relatively low dose influences (1 × 10^13^ ions/cm^2^), the minimal detectable optical power can be improved from − 33.2 to − 63.1 dBm at the wavelength of 1550 nm. Moreover, the photo-response at the same optical power (− 10 dBm) can be enhanced by approximately 18.8 dB (with a dose of 1 × 10^13^ ions/cm^2^). The transmittance spectra from 1200 to 1800 nm, with and without helium-ion implantation, stay almost unchanged, confirming that the transparency of the photodetector is not noticeably affected.

## Experiments

**Fig. 1:**
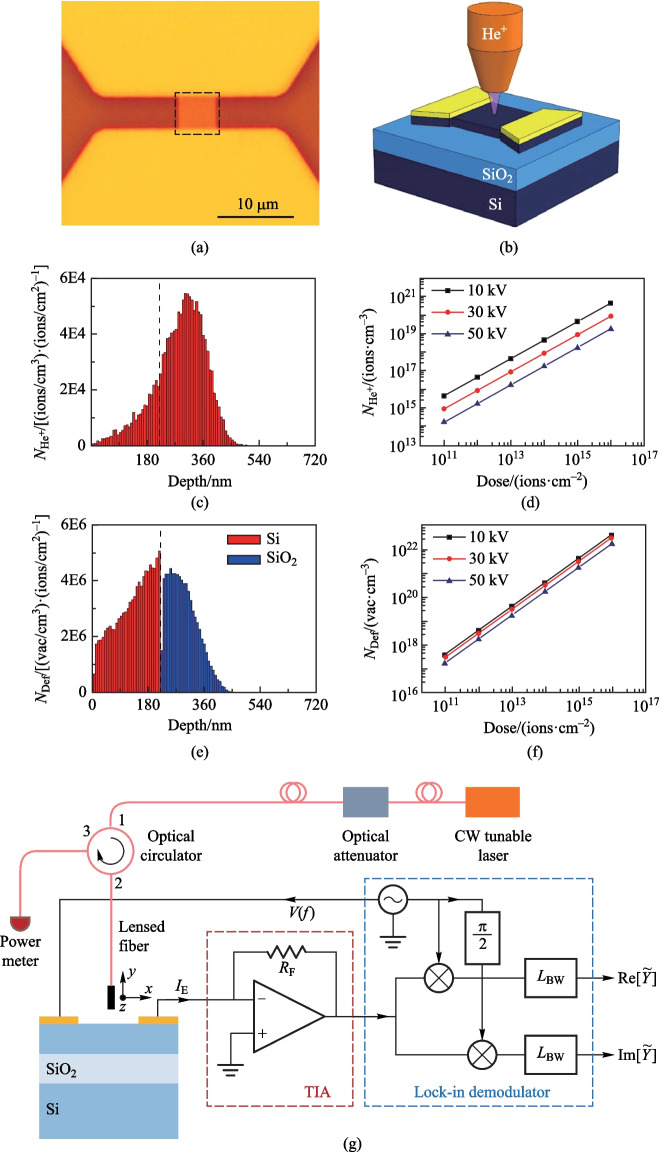
Helium-ion implantation and device-testing setup.** a** Optical micrograph of the silicon sub-bandgap photodetector. The dashed box shows the area with helium-ion implantation. The area was 6.5 μm by 6.5 μm, slightly larger than the photosensitive area, 5 μm by 5 μm, of the photodetector. **b** Schematic drawing of locally implanting helium ions to the silicon sub-bandgap photodetector by using a helium-ion microscope. **c** Simulated helium-ion distribution per unit dose in the top silicon and silicon oxide. The accelerating voltage was 30 kV. **d** Monte–Carlo simulation of the volume density of the helium ions implanted in top silicon of the SOI wafer, as a function of the dose and at three accelerating voltage, 10, 30, and 50 kV. **e** Simulated defect distribution per unit dose in the top silicon and silicon oxide. The accelerating voltage was 30 kV. **f** Monte–Carlo simulation of the volume density of the vacancy generated in top silicon of the SOI wafer, as a function of the dose and at three accelerating voltages, 10, 30, and 50 kV. **g** Schematics of the experimental setup

The silicon sub-bandgap photodetector was fabricated on a silicon-on-insulator (SOI) wafer using the same recipe as reported in Ref. [5], with an additional step to strip the residual photoresist on top of the photodetector by immersing the chip in hot *N*-methyl pyrrolidone (NMP) at 95 ℃ for 30 min. The photosensitive area of the photodetector was 5 μm by 5 μm, and the thickness of the top silicon was 220 nm. The optical micrograph of the photodetector is shown in Fig. 1a. The region in the dashed box was 6.5 μm by 6.5 μm and was the region for helium-ion implantation. Figure 1b presents the schematic drawing of helium-ion implantation, using a helium-ion microscope. In the process of implantation, the helium-ion beam scanned over the area in the dashed box with a step size of 0.25 nm. We precisely controlled the dose by precisely controlling the beam current of the helium-ion microscope and writing/implantation time. The helium-ion microscope we used had a beam gating time precision of 0.1 μs, and we used a beam current of 0.5 pA.

We simulated the distribution and density of helium ions and defects generated by ion bombardment, using the software SRIM, which was based on the Monte-Carlo method [18]. Figure 1c and e present the distribution of helium ions and defects per unit dose, respectively, in both the top silicon and silicon oxide layers. The accelerating voltage was 30 kV. Figure 1d and f present ${N}_{{\text{H}}{\text{e}}^{+}}$ and ${N}_{\mathrm{Def}}$, as a function of the dose, at three accelerating voltages, 10, 30, and 50 kV. Note that for the same dose, increasing the accelerating voltage decreased both ${N}_{{\text{H}}{\text{e}}^{+}}$ and ${N}_{\mathrm{Def}}$. In our experiment, we kept the doses low to avoid material swelling [8] that could affect the optical properties of the photodetectors. We used a 30 kV accelerating voltage and doses of 1 × 10^13^, 5 × 10^13^, 1 × 10^14^ and 1 × 10^15^ ions/cm^2^ in the experiment and ${N}_{{\text{H}}{\text{e}}^{+}}$ values estimated by the simulation were 8.7 × 10^16^, 4.4 × 10^17^, 8.7 × 10^17^ and 8.7 × 10^18^ cm^−3^ for these four doses, respectively, and ${N}_{\mathrm{Def}}$ values were estimated to be 3.1 × 10^19^, 1.6 × 10^20^, 3.1 × 10^20^ and 3.1 × 10^21^ cm^−3^, respectively.

The experimental setup, as schematically presented in Fig. 1g, and the method to characterize the silicon sub-bandgap photodetectors were almost identical to those reported in Ref. [5]. Very briefly, we used a lensed fiber with a spot mode field diameter of 2 μm for vertical laser illumination of the surface of the photosensitive area of the silicon sub-bandgap photodetector; the optical power to the silicon sub-bandgap photodetector could be adjusted by a variable optical attenuator. The silicon sub-bandgap photodetector was driven by an AC voltage, $V(f)$, from a lock-in amplifier, and the current signal from silicon sub-bandgap photodetector, ${I}_{\mathrm{E}}$, was changed into a voltage signal, ${V}_{\mathrm{d}}$, by a transimpedance amplifier (TIA), and read out by the lock-in amplifier. The measured admittance of silicon sub-bandgap photodetector can be expressed as

$$\tilde{Y} = I_{{\text{E}}} /V\left( f \right) = \left( {V_{{\text{d}}} /G} \right)/V\left( f \right) = Y{\text{e}}^{{{\text{j}}\theta }} ,$$

where $G$ is the gain of TIA, $\mathrm{j}=\sqrt{-1}$, $\theta$ is the phase. In all measurements thereafter, the amplitude of the driven voltage is $\mathrm{1~V}$, and $G={10}^{7}~\Omega$. To make comparisons, five cases, without helium-ion implantation, with implanting doses of 1 × 10^13^, 5 × 10^13^, 1 × 10^14^, and 1 × 10^15^ ions/cm^2^, were investigated.

## Results and discussion

**Fig. 2:**
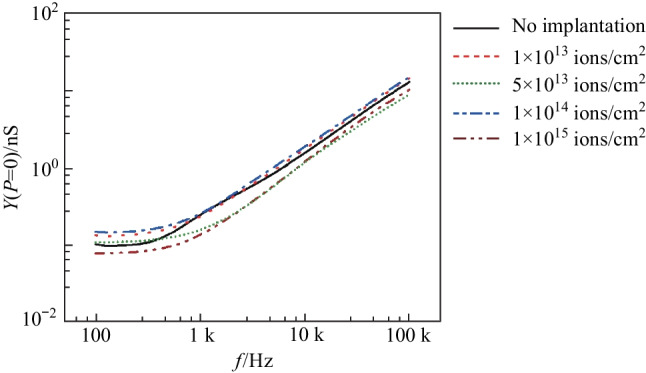
Measured admittance, *Y*, of the photodetector as a function of driven frequency, *f*, without illumination. Five cases were investigated: without helium-ion implantation, and with implanting doses of 1 × 10^13^, 5 × 10^13^, 1 × 10^14^, and 1 × 10^15^ ions/cm^2^

The first measurement was of the admittance in the dark, $Y(P=0)$. Figure 2 presents $Y(P=0)$ as a function of driven frequency, $f$. The results show that implantation did not affect $Y(P=0)$ except for very minor differences in the $Y-f$ curves. The interpretation was that the defect states generated by ion implantation made little contribution to the current transport when the photodetector was in the dark.

**Fig. 3:**
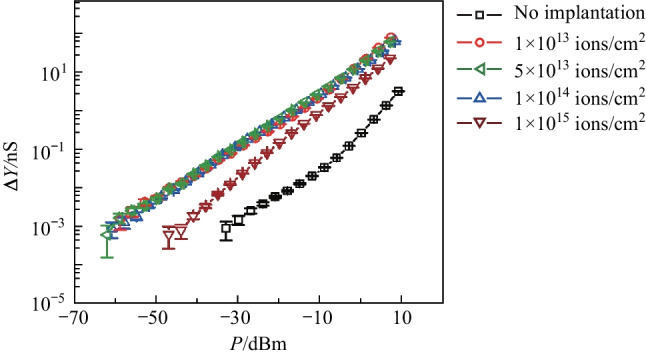
Measured photo-response, Δ*Y*, of the photodetector as a function of illuminating optical power, *P*. The driven frequency, *f*, was 521 Hz. Five cases were investigated: without helium-ion implantation, and with implanting doses of 1 × 10^13^, 5 × 10^13^, 1 × 10^14^, and 1 × 10^15^ ions/cm^2^

The second measurement was of the light-induced admittance variation, $\Delta Y(P)=|Y\left(P\right)|-|Y\left(P=0\right)|$, as a function of incident optical power, $P$. In this measurement, $f=521 \, \mathrm{ Hz}$, and the lock-in bandwidth $\Delta f=3 \, \mathrm{ Hz}$. Figure 3 presents the results and shows a dramatic enhancement of $\Delta Y$ by helium-ion implantation. The error bar associated with each data point shows the standard deviation of 4499 measurements. For example, from Fig. 3, when the optical power is − 10 dBm, $\Delta Y$ of the sub-bandgap photodetector without helium ions implanted is 0.03 nS, $\Delta Y$ of sub-bandgap photodetectors with an implantation dose of 1 × 10^13^, 5 × 10^13^, 1 × 10^14^, and 1 × 10^15^ ions/cm^2^ is 2.3, 3.0, 2.5 and 0.9 nS, respectively. The enhancement effect for the doses, 1 × 10^13^, 5 × 10^13^ and 1 × 10^14^ ions/cm^2^, is similar as indicated by the three almost identical curves of $\Delta Y(P)$. In particular, for the dose of 1 × 10^13^ ions/cm^2^, the enhancement of the photo-response is 10log(2.3/0.03)=18.8 dB. We attribute this enhancement to the increase of the defect states within the bandgap, and therefore, the sub-bandgap optical absorption was enhanced. However, further increasing the implantation dose to 1 × 10^15^ ions/cm^2^ weakened the enhancement effect.

**Fig. 4:**
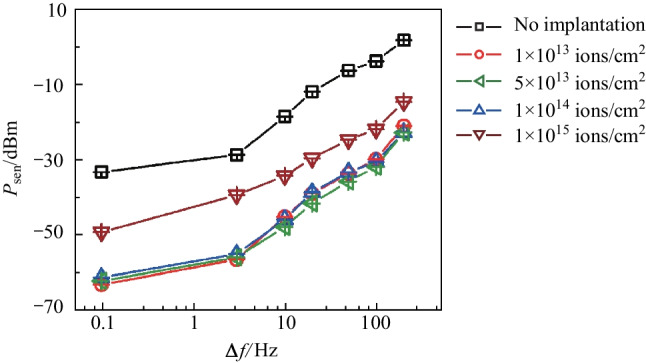
Measured minimal detectable optical ${P}_{\mathrm{sen}}$ as a function of lock-in bandwidth, $\Delta f$. Five cases were investigated: without helium-ion implantation, and with implanting doses of 1 × 10^13^, 5 × 10^13^, 1 × 10^14^, and 1 × 10^15^ ions/cm^2^

**Fig. 5:**
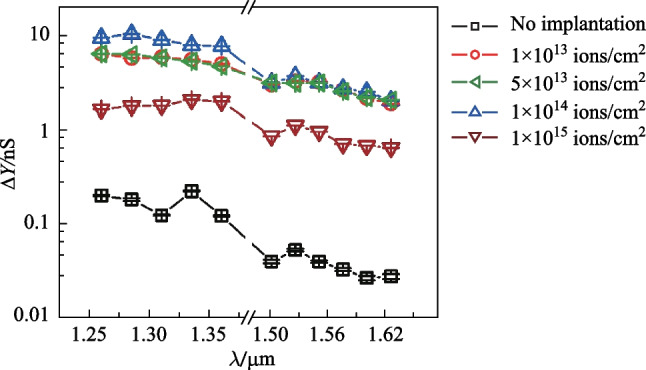
Measured photo-response spectra of the photodetector. Five cases were investigated: without helium-ion implantation, and with implanting doses of 1 × 10^13^, 5 × 10^13^, 1 × 10^14^, and 1 × 10^15^ ions/cm^2^. The power of incident light was − 10 dBm

The third measurement was the minimal detectable optical power, ${P}_{\mathrm{sen}}$, at different lock-in bandwidth, $\Delta f$, as shown in Fig. 4. ${P}_{\mathrm{sen}}$ is defined by $\Delta Y({P}_{\mathrm{sen}})=6\sigma$, where $\sigma$ is the standard deviation of $|Y\left(P=0\right)|$, representing the amplitude of the noise. ${P}_{\mathrm{sen}}$ characterizes the sensitivity of the sub-bandgap photodetector. In our experiment, $\sigma$ stayed almost unchanged without and with helium-ion implantation. For the five cases investigated, the overall trend that ${P}_{\mathrm{sen}}$ increased as $\Delta f$ increased was the same [5]. However, because helium-ion implantation enhanced the photo-response, as shown in Figs. 3 and 5, the photodetectors became more sensitive, i.e., ${P}_{\mathrm{sen}}$ decreased, compared with $\mathrm{its~value }$ without helium-ion implantation. Specifically, when $\Delta f=0.1 \, \mathrm{Hz}$, ${P}_{\mathrm{sen}}$ of the silicon sub-bandgap photodetector without helium ions implanted was − 33.2 dBm, and ${P}_{\mathrm{sen}}$ values of the silicon sub-bandgap photodetector implanted with helium ions at doses of 1 × 10^13^, 5 × 10^13^, 1 × 10^14^, and 1 × 10^15^ ions/cm^2^ were − 63.1, − 62.1, − 61.2, and − 49.2 dBm, respectively. The sensitivity was improved by 29.9, 28.9, 28, and 16 dB, respectively.

The fourth measurement was the spectral response. Two tunable semiconductor lasers were used, one ranging from 1260 to 1360 nm and the other ranging from 1500 to 1630 nm. For these measurements, we kept the optical power to be − 10 dBm. As presented in Fig. 5, in these two bands, helium-ion implantation dramatically increased Δ*Y*, showing this enhancement was broadband.

**Fig. 6:**
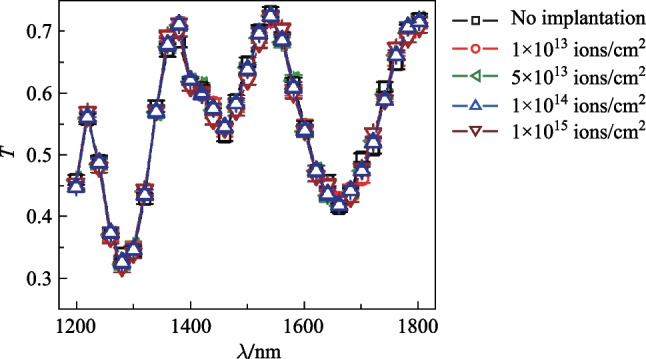
Measured optical transmittance spectra of the photodetector. Five cases were investigated: without helium-ion implantation, and with implanting doses of 1 × 10^13^, 5 × 10^13^, 1 × 10^14^, and 1 × 10^15^ ions/cm^2^

Finally, we measured the spectra (from 1200 to 1800 nm) of optical transmittance of the photosensitive region with and without helium implantation. To make these measurements, a SuperK with a monochromator was used as a tunable light source. A fiber focuser with an expanded mode field diameter of 4.3 μm was used to vertically illuminate a larger photosensitive region, 50 μm by 50 μm. The expansion of the mode size of the incident beam was necessary because otherwise the beam would have expanded too fast as it would have propagated such that the size of the beam would have been larger than the photosensitive region of the optical power meter used in our experimental setup. The optical transmittance was taken as ${T=P}_{1}/{P}_{2}$, where ${P}_{1}$ is the optical power transmitted through and ${P}_{2}$ is the incident power illuminating the silicon sub-bandgap photodetector. Each error bar in Fig. 6 represents the standard deviation of 50 measurements. The figure presents the results, showing that the spectra of the transmittance without helium-ion implantation and with implantation highly overlapped. The results mean that helium-ion implantation did not affect the transparency. We note in the figure that *T* maximizes at the wavelength of 1540 nm, and the maximum value of *T* is 73%. By appropriately coating anti-reflection coatings on the back of the photodetector, we can further increase *T* in this wavelength range.

We can now compare the advantages and disadvantages of helium ion implantation over other techniques to enhance the photodetection. Researchers enhance photodetection by optimizing the device structures and/or modifying the material properties. One example of a method for optimizing the device structures is to integrate the detectors with resonators [19]. This category of technique is typically used for enhancing the photodetection in a certain narrow band. In comparison, helium-ion implantation can enhance silicon sub-bandgap photodetection across a broad wavelength range, which is one advantage of this technique. On the other hand, use of the helium-ion microscope to implant the ions is a serial process and is only suitable for photodetectors with small photosensitive area. Moreover, the depth of implantation is limited, making this technique unsuitable for the cases that need to modify the material properties deeper in the photodetectors.

## Conclusion

In conclusion, we have enhanced the photo-response of silicon sub-bandgap photodetectors by implanting helium ions with relatively low dose influences. With an implantation dose of 1 × 10^13^ ions/cm^2^, the minimal detectable optical power was improved from − 33.2 to − 63.1 dBm, or, by 29.9 dB, at the wavelength of 1550 nm, and the photo-response at the same optical power (− 10 dBm) was enhanced by approximately 18.8 dB. We measured the transmittance spectra and observed no changes by helium-ion implantation. We attribute the enhancement of the photo-response to the increase of the defect states within the bandgap, which further increases the sub-bandgap optical absorption. This absorption, although being enhanced, still stays low ensuring the transparency of the photodetector. Our work provides a method for strategically modifying the intrinsic trade-off between transparency and strong photo-responses for this type of photodetectors.

## Data Availability

The data that support the findings of this study are available from the corresponding author, upon reasonable request.
